# Network Clustering Revealed the Systemic Alterations of Mitochondrial Protein Expression

**DOI:** 10.1371/journal.pcbi.1002093

**Published:** 2011-06-30

**Authors:** Jouhyun Jeon, Jae Hoon Jeong, Je-Hyun Baek, Hyun-Jung Koo, Wook-Ha Park, Jae-Seong Yang, Myeong-Hee Yu, Sanguk Kim, Youngmi Kim Pak

**Affiliations:** 1 Division of Molecular and Life Science, School of Interdisciplinary Bioscience and Bioengineering, Pohang University of Science and Technology, Pohang, Korea; 2 Department of Life and Nanopharmaceutical Sciences, Kyung Hee University, Seoul, Korea; 3 Functional Proteomics Center, Korea Institute of Science and Technology, Seoul, Korea; 4 Department of Physiology, College of Medicine, Kyung Hee University, Seoul, Korea; 5 Division of ITCE engineering, Pohang University of Science and Technology, Pohang, Korea; University of Chicago, United States of America

## Abstract

The mitochondrial protein repertoire varies depending on the cellular state. Protein component modifications caused by mitochondrial DNA (*mt*DNA) depletion are related to a wide range of human diseases; however, little is known about how nuclear-encoded mitochondrial proteins (*mt* proteome) changes under such dysfunctional states. In this study, we investigated the systemic alterations of *mt*DNA-depleted (ρ^0^) mitochondria by using network analysis of gene expression data. By modularizing the quantified proteomics data into protein functional networks, systemic properties of mitochondrial dysfunction were analyzed. We discovered that up-regulated and down-regulated proteins were organized into two predominant subnetworks that exhibited distinct biological processes. The down-regulated network modules are involved in typical mitochondrial functions, while up-regulated proteins are responsible for *mt*DNA repair and regulation of *mt* protein expression and transport. Furthermore, comparisons of proteome and transcriptome data revealed that ρ^0^ cells attempted to compensate for *mt*DNA depletion by modulating the coordinated expression/transport of *mt* proteins. Our results demonstrate that *mt* protein composition changed to remodel the functional organization of mitochondrial protein networks in response to dysfunctional cellular states. Human *mt* protein functional networks provide a framework for understanding how cells respond to mitochondrial dysfunctions.

## Author Summary

Mitochondria are dynamic organelles that are essential for energy production and cellular processes in eukaryotic cells, and their functional failure is a major cause of age-associated degenerative diseases. To meet the specific needs of different cellular states, mitochondrial protein repertoires are adjusted. It is critical to characterize the systemic alterations of mitochondria to different cellular states to understand the dynamic organization of mitochondrial systems. In this study, we modularized the quantified proteomics data into protein functional networks to characterize gene expression changes under dysfunctional mitochondrial conditions. Our results demonstrate that mitochondrial protein repertoires changed to compensate for dysfunctional cellular states by reorganizing mitochondrial protein functional network. Through network clustering analysis, we discovered that cells respond to pathological conditions by modulating the coordinated expression/transport of mitochondrial proteins. Network analysis of *mt* proteins can advance our understanding of dysfunctional mitochondrial systems and elucidate the candidate *mt* proteins involved in human mitochondrial diseases.

## Introduction

Proteomics and expression profiling have been widely applied to understand the cellular processes [1]. Proteins with significant changes in expression have particular interest as markers for various diseases and cellular phenotypes [2]. While there are useful information encoded in the list of differentially expressed proteins, identifying the molecular mechanism of cellular processes from the long list of candidate proteins is challenging [3]. Data-integrative approaches have been successfully applied to address the challenge of interpreting lists of differentially expressed proteins by mapping them onto the protein functional network [4], [5].

A protein functional network describes the functional and physical associations among proteins and provides a framework for understanding how individual protein work together to perform critical cellular functions and how protein compositions respond to changes in cellular environments [6]. Proteins rarely act alone but rather interact with other proteins and comprise specific functional modules in the network [7]. Functional module is a group of proteins which are related by one or more cellular and genetic interactions such as co-regulation, co-expression, and the member of a biological pathway or a protein complex [8]. Such module is the building block of cellular organization and carries out unique biological process [9]. Therefore, understanding the modular structure of protein functional network should be useful for characterizing the dynamic organization of cellular systems.

In eukaryotic cells, mitochondria are involved in many cellular processes including energy production, apoptosis, ion homeostasis, and the metabolism of glucose, lipids, and amino acids [10]. Although mitochondria possess their own DNA, it is estimated that at least 98% of the 1,500–2,000 mitochondrial proteins are encoded by nuclear genes and shuttled posttranslationally into the mitochondria [11], [12]. In addition, the majority of human *mt* disorders are known to be related with nuclear genome defects [13]. Thus, compiling a comprehensive list of *mt* proteins is essential to understand mitochondrial biogenesis and pathology. Large-scale approaches such as mass spectrometry (MS)-based proteomics [14], epitope tagging combined with microscopy [15], genome-wide predictions of protein subcellular localizations [16], and comparative genomics analyses [17], [18], [19] have revealed the localization of the *mt* protein inventory.

The *mt* protein inventory is dynamically changed depending on the cellular state, tissue type, and species [11], [20]. For example, the compositions of *mt* proteins are different across various tissues and organs in mice [21], [22] and changed by fermentation states in yeast [18] or environmental stresses in plants [23]. Additionally, in humans, dynamic changes in the *mt* proteome affect the functional organizations of *mt* proteins and disease susceptibilities [24]. Genetic or biochemical abnormalities in mitochondria caused by complete or partial *mt*DNA depletion have been linked to a wide range of human diseases including metabolic syndrome, neurodegenerative disorders, mitochondrial myopathy, and cancer [12], [25]. We have previously reported that *mt*DNA depletion can lead to impairments of glucose metabolism [26], insulin signaling [27], and apoptosis [28]. However, the changes of the *mt* protein functional network in response to *mt* dysregulation remain to be revealed.

Here, we investigated the systemic alterations of human *mt* protein functional network under normal and dysfunctional *mt* states through a data-integrative computational biology approach and quantitative proteomic analysis. Specifically, a systematic data-integrative analysis was devised to evaluate the reliability of *mt* proteomics data and cluster the identified proteins into the modules of *mt* protein functional network. Our results revealed that human *mt* proteins comprise specific network modules to control unique biological processes in cells exposed to dysfunctional *mt* states. Furthermore, network clustering suggests that cells respond to pathological conditions by modulating the coordinated expression and transport of mitochondrial proteins. We believe that our results may provide critical information to gain better understanding of mitochondria function in the cell.

## Results

### Systemic properties of mitochondria under an *mt*DNA-depleted dysfunctional state

We investigated the systemic alterations of mitochondria by using network analysis of quantified proteomics data. A data-integrative approach was devised to select reliable *mt* proteins for network analysis.

First, we applied the isotope-coded affinity tag (cICAT) quantitative analysis [29] to compare protein abundances in mitochondria isolated from normal (ρ^+^) and *mt*DNA-depleted (ρ^0^) osteosarcoma cells. *Mt*DNA-depleted (ρ^0^) osteosarcoma cell has been used as an important tool to investigate dysfunctional mitochondria. The ρ^0^ cell was established by long-term treatment of ethidium bromide (EtBr) which intercalated into *mt*DNA without any detectable effect on nuclear DNA division [30] and led to the inhibition of *mt*DNA replication and transcription [31]. Thus, we examined a set of nuclear-encoded *mt* proteins as *mt* proteome. To quantify protein abundance ratios of *mt* proteins in ρ^0^ versus ρ^+^ mitochondria, we labeled *mt* proteins with cICAT containing light (^12^C) and heavy (^13^C) isotope signatures, which react with thiol groups of cysteines in proteins. The experimental scheme is summarized in Figure S1 (see Materials and Methods for details). From the cICAT analysis, we identified 1,121 proteins (Table S1). According to the *mt* protein abundance ratios (ρ^0^/ρ^+^), we classified all proteins into three classes: up-regulated, down-regulated, and not significantly changed proteins in ρ^0^ mitochondria. The number of up-regulated proteins with ρ^0^/ρ^+^≥1.5 was 201, while the number of down-regulated proteins with ρ^0^/ρ^+^≤0.67 was 313.The thresholds of 1.5 represents the 1.5-fold increase, whereas the threshold of 0.67 represents 1.5-fold decrease under dysfunctional ρ^0^ state. Meanwhile, 607 not significantly changed proteins were present in both ρ^0^ and ρ^+^ mitochondria with similar abundances (0.67<ρ^0^/ρ^+^<1.5).

**Figure 1:**
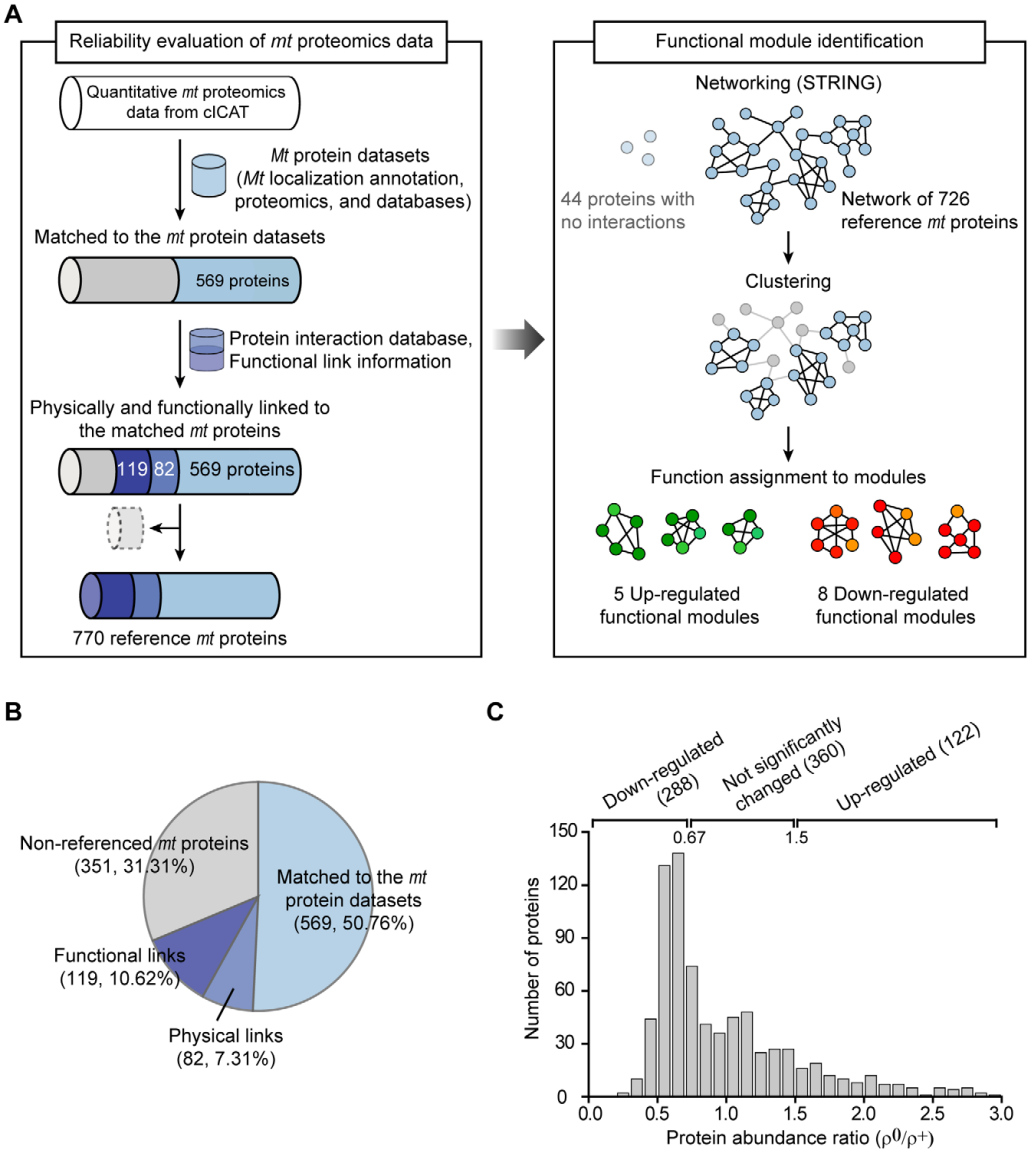
Analysis of the human *mt* proteomics data. (A) Reliability evaluation of *mt* proteomics data and functional module identification. (B) Compositions of the *mt* proteins from the cICAT proteomics data. (C) Distributions of protein abundance ratios (ρ^0^/ρ^+^) in reliable *mt* proteins. Down-regulated, not significantly changed, and up-regulated proteins were shown.

Next, the 1,121 proteins identified via cICAT analysis were evaluated by a systematic data-integrative approach to select more reliable *mt* proteins (Figure 1A). First, we examined the Gene Ontology (GO) cellular component annotation for the identified proteins and compiled 13 reference *mt* protein datasets from seven *mt* proteins databases and six *mt* proteomics datasets (see Materials and Methods for details). In total, 569 out of 1,121 proteins (50.76%) were annotated as mitochondrial proteins and observed from at least one reference *mt* protein dataset (Figure 1B). Then, we assessed the physical and functional links of reference *mt* proteins based on the assumption that protein pairs that interact or share similar functions tend to cluster within the same subcellular organelle [15]. A total of 201 proteins were physically (82 proteins, 7.31%) or functionally (119 proteins, 10.62%) linked to the 569 reference *mt* proteins (Figure 1B). Physical link represents protein-protein interaction. Meanwhile, functional link represents a relationship between two proteins if they shared a substrate in a metabolic pathway, co-expressed, co-regulated, or involved in the same protein complex. We listed these 770 *mt* proteins (569+82+119) in Table S1 as a reliable *mt* protein dataset. Among the 770 *mt* proteins, the numbers of down-regulated, up-regulated, and not significantly changed proteins were 288, 122, and 360, respectively (Figure 1C). The remaining proteins (351 proteins, 31.31%) were assigned as non-referenced *mt* proteins that might be either novel *mt* proteins or proteins with localizations that were affected by *mt*DNA depletion.

**Figure 2:**
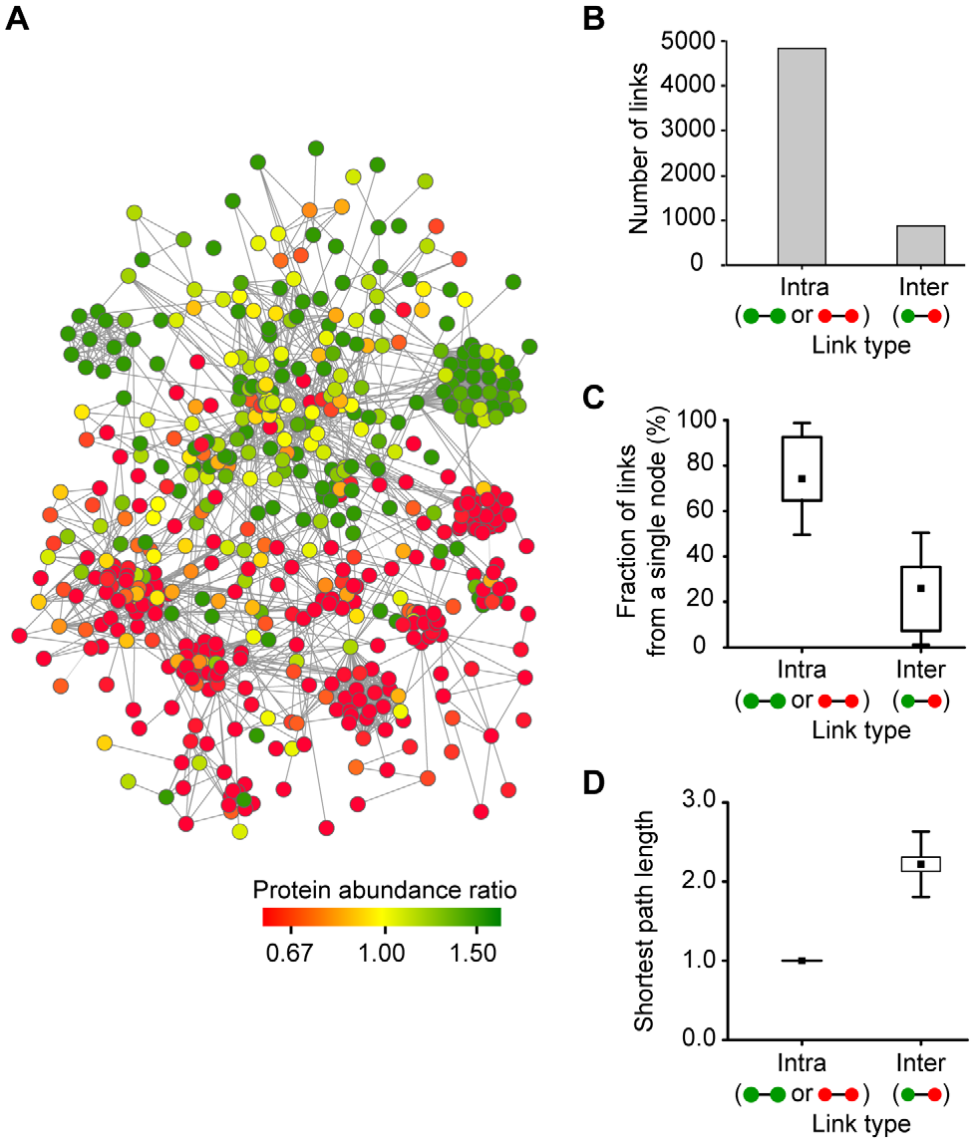
Human *mt* protein functional network. (A) Global functional network of human *mt* proteins. Nodes are color coded according to the ρ^0^/ρ^+^ ratio. Green and red nodes represent up-regulated *mt* proteins and down-regulated *mt* proteins under the dysfunctional ρ^0^ state, respectively. (B) Number of links according to the link types. An intraregulatory link is a link between proteins with the same regulatory pattern: up- and up-regulated or down- and down-regulated proteins. An interregulatory link is a link between up- and down-regulated proteins. (C) The fraction of link types per single protein. (D) The shortest path length according to the link types.

Different cellular states change the expression and localization of proteins targeting mitochondria [22]. To analyze the systemic properties of the *mt* proteome under the dysfunctional ρ^0^ state, we constructed a network of reliable *mt* proteins. By using information about the physical and functional links of these proteins, we could map 726 out of 770 reliable *mt* proteins into a major network (right panel in Figure 1A; see Materials and Methods for details). The remaining 44 proteins disconnected from the major network were excluded from further network analysis. We discovered that the network is divided into two prominent subnetworks of up-regulated (green) and down-regulated proteins (red) based on abundance ratios (Figure 2A). Interestingly, more links were made within up-regulated or down-regulated proteins (intraregulatory links) than between up- and down-regulated proteins (interregulatory links). Among the 5,713 links in the network, the majority (4,854 links, 84.96%) were intraregulatory links (Figure 2B). When we measured the fraction of links per protein, intraregulatory links were 3-fold more common than interregulatory links (Whitney-Mann U test, *p*-value = 7.55×10^−78^; Figure 2C). Furthermore, the shortest path length of proteins connected within intraregulatory links was smaller than that of proteins connected within interregulatory links (Whitney-Mann U test, *p*-value = 7.55×10^−156^; Figure 2D). Shortest path length is the minimum number of links connecting one protein to another protein in the network, thus a smaller shortest path length implies that two proteins are more closely related [6]. This result indicates that up-regulated and down-regulated proteins tend to cluster themselves and might have distinct functional roles in the protein functional network.

To examine whether up-regulated and down-regulated proteins participate in different biological processes, we examined the enriched function of proteins according to abundance ratios. We discovered that significant biases exist between the two groups when they were classified by GO biological process terms (Table S2). Functions of up-regulated proteins were involved in mRNA metabolism and cytosolic ribosome-mediated translation, whereas down-regulated proteins were involved in *mt* ribosome-mediated translation, oxidative phosphorylation, and the TCA cycle (*p*-value <1×10^−3^).

### Functional composition of *mt* proteome changes under the dysfunctional *mt* state

**Figure 3:**
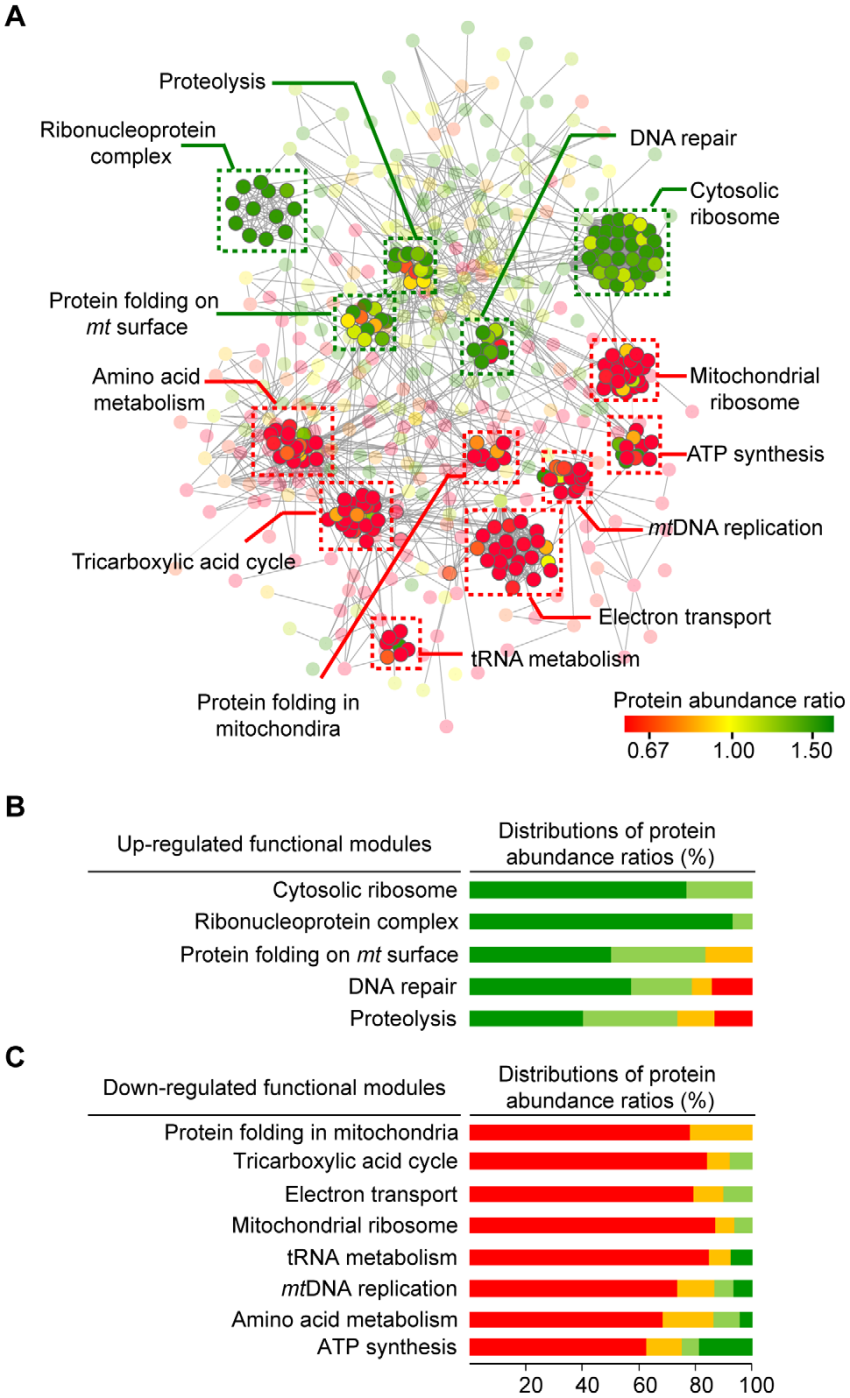
Thirteen significantly changed functional modules under *mt*DNA-depleted dysfunctional state. (A) Five up-regulated functional modules (green boxes) and eight down-regulated functional modules (red boxes) were shown. (B) Distributions of protein abundance ratios in five up-regulated functional modules. (C) Distributions of protein abundance ratios in eight down-regulated functional modules.

To identify significantly altered functional groups under the dysfunctional ρ^0^ state, we organized *mt* proteins into functional modules (right panel in Figure 1A). We identified modules by examining whether a group of proteins was physically connected or functionally linked. We used hierarchical average-linkage clustering with the STRING confidence scores as a similarity measure (Materials and Methods for details). Then, we assigned the biological function to the module by examining the representative functional annotations from proteins in each module. Finally, we identified 13 functional modules that were enriched either within up-regulated or down-regulated protein groups (*p*-value <0.01; Table S3). As shown in Figure 3A, five functional groups were up-regulated (shown in green boxes), whereas eight functional groups were down-regulated (shown in red boxes) under the dysfunctional ρ^0^ state. The five up-regulated groups contain 59 out of 89 (66.29%) up-regulated proteins. They are linked to the function of cytosolic ribosome, ribonucleoprotein complex, protein folding on *mt* surface, DNA repair, and proteolysis, which are associated with the regulation of *mt* protein expression in response to *mt*DNA damage (Figure 3B). Conversely, the eight down-regulated groups include 138 out of 175 (78.86%) down-regulated proteins. They were associated with basal *mt* functions, such as mitochondrial energy production, metabolism, and protein folding in mitochondria (Figure 3C). Our result suggests that the expression control of *mt* proteins that are involved in different functional modules is regulated separately under dysfunctional *mt* states.

### Expression profile analysis of *mt* protein and mRNA under a dysfunctional *mt* state

**Figure 4:**
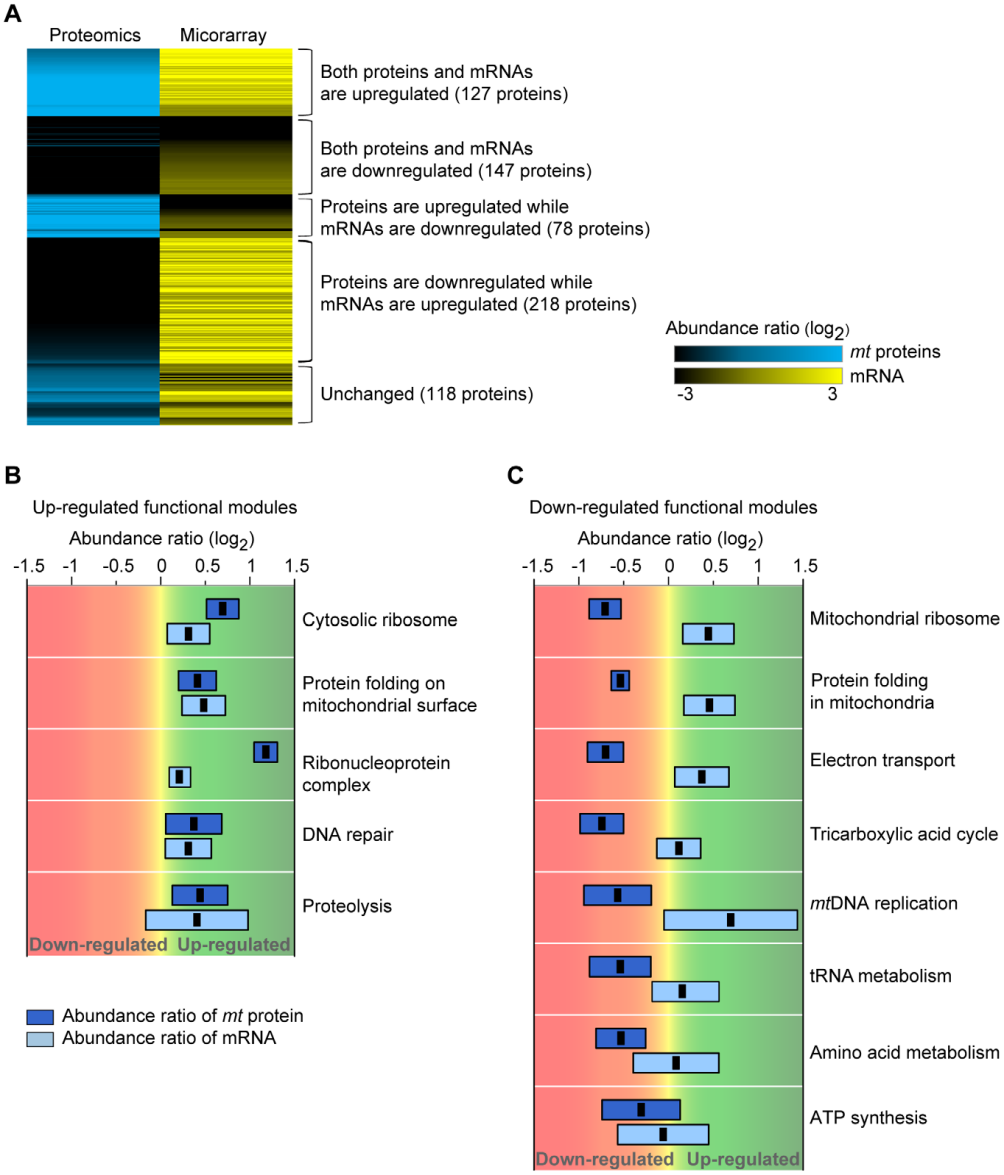
Mitochondrial proteome-transcriptome profiles in the dysfunctional ρ^0^ state. (A) Expression patterns of *mt* proteins and mRNAs. As abundance ratios of proteins and mRNAs increased, the colors changed to blue (*mt* protein) and yellow (mRNA). (B) Box-plots of protein and mRNA abundance ratios for the up-regulated functional modules. Abundance ratios of protein and mRNA were colored as blue and light blue, respectively. The error bars indicate the standard deviations of protein and mRNA abundance ratios. The black dots represent the average protein and mRNA abundance ratios. (C) Box-plots of protein and mRNA abundance ratios for the down-regulated functional modules.

Eukaryotic cells can monitor and respond to changes in *mt* conditions through alterations of nuclear gene expression [32]. To understand how cells respond to the dysfunctional ρ^0^ state to facilitate survival, we analyzed the expression profiles of *mt* proteins and mRNAs. We found that 40% of *mt* proteins (274 = 127+147) exhibited a positive correlation between protein and mRNA abundances, while 43% of *mt* proteins (296 = 78+218) exhibited a negative correlation between protein and mRNA abundances (Figure 4A). By using the k-means clustering algorithm, the mRNA and protein-expression profiles were divided into five groups depending on the abundance ratios of *mt* protein-mRNA: up-up (127), down-down (147), up-down (78), down-up (218), and unchanged (118) groups (Figure 4A and Table S4 for protein list). We found that up-regulated and down-regulated functional modules exhibited distinctive patterns in expression profiles (Figure 4B and C; details in Table S3). Specifically, both protein and mRNA abundances increased in the five up-regulated functional modules (Figure 4B). Conversely, mRNA abundances increased, but *mt* protein levels were decreased in the eight down-regulated functional modules (Figure 4C). The up-regulated functional modules were involved in *mt*DNA maintenance and control of *mt* protein transport. The down-regulated functional modules were associated with mitochondrial energy production and metabolism. These results suggest that cells actively modulate the expression and transport of *mt* proteins depending on the functions necessary to survival under pathological conditions.

### Validation of the abundance and localization of *mt* proteins

**Figure 5:**
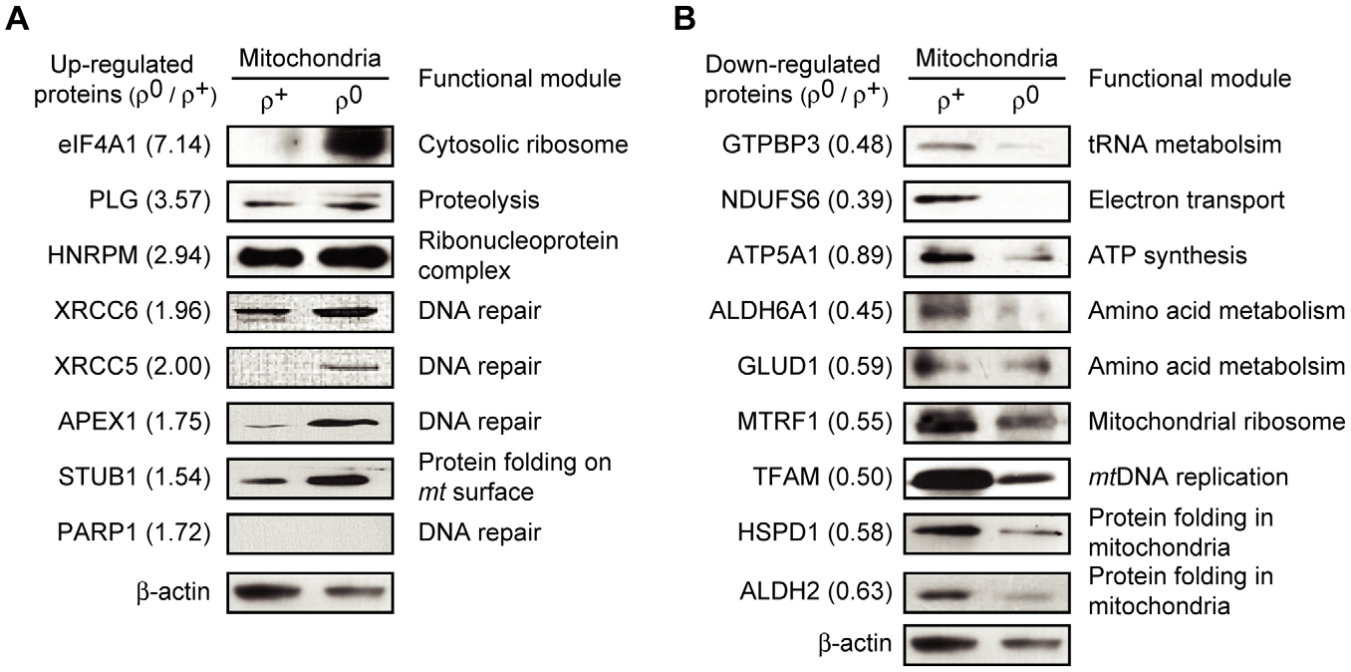
Validations of expression changes of *mt* proteins. Expression of (A) eight up-regulated proteins and (B) nine down-regulated proteins from the different functional modules were examined. Mitochondrial lysates (10 µg) were resolved using 12% SDS-PAGE and analyzed by western blot. Numbers in parenthesis are the protein abundance ratios. β-actin served as a loading control.

To validate protein abundance ratios measured by cICAT, we performed western blot analysis by using isolated ρ^+^ and ρ^0^ mitochondria and confirmed that the tested protein abundances were largely consistent with those measured by cICAT (Figure 5). We selected eight up-regulated and nine down-regulated proteins from each functional module, of which antibodies were commercially available. We found that the levels of seven out of the eight up-regulated proteins were increased in ρ^0^ mitochondria (Figure 5A). The up-regulated proteins were eIF4A1, PLG, HNRPM, XRCC6 (Ku70), XRCC5 (Ku80), APEX1, and STUB1. Likewise, the levels of all tested down-regulated proteins were decreased in ρ^0^ mitochondria, consistent with the cICAT quantifications (Figure 5B). The down-regulated proteins were GTPBP3, NDUFS6, ATP5A1, ALDH6A1, GLUD1, MTRF1, TFAM, HSPD1 (HSP60), and ALDH2. The change of PARP1 levels was not detected by western blot. Additionally, the reliability of the protein expression patterns of cICAT was further confirmed by comparing the expression patterns of previously reported 2DE proteomic analyses [33]. Thirty of the 33 *mt* proteins (90.91%) identified from the 2DE proteomic analyses showed similar expression patterns compared to those obtained from the cICAT analysis (Figure S2). The difference between protein expression patterns of cICAT and 2DE was insignificant (*p*-value was only 0.08).

**Figure 6:**
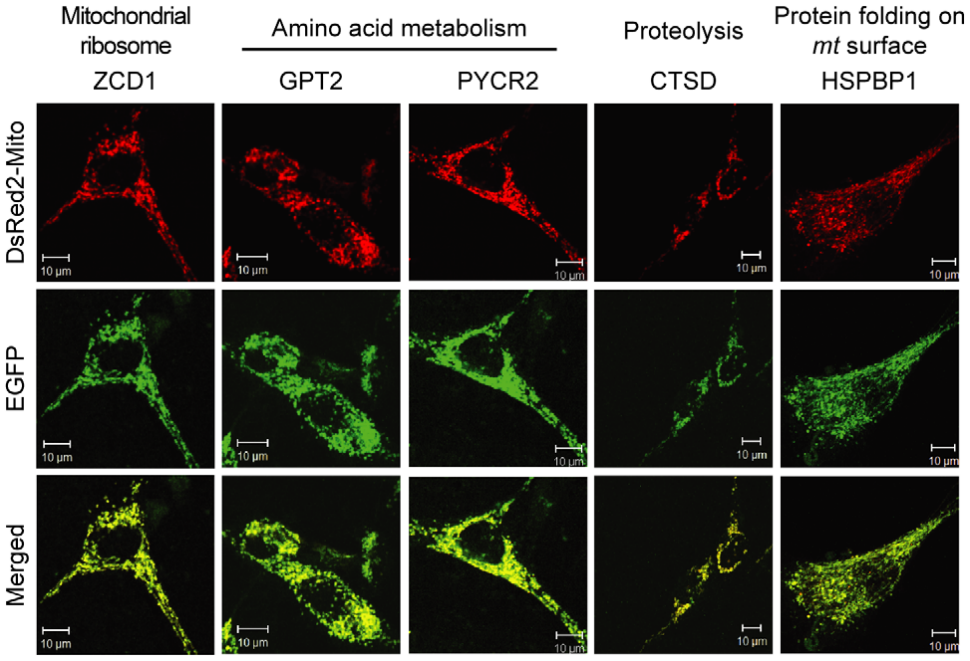
Validating mitochondrial localizations of *mt* proteins. SK-Hep1 cells expressing DsRed2-mito were transfected with GFP-hybrid plasmids of ZCD1, GPT2, PYCR2, CTSD, and HSPBP1. The transfected cells were fixed, mounted, and imaged using a confocal microscope. The functional module of each protein is presented on the top. Merged images of EGFP and DsRed signals represent the *mt* localization of the proteins. Scale bar = 10 µm.

To verify the localizations of the identified *mt* proteins, we selected five proteins and cloned their cDNAs to express GFP-hybrid proteins. We examined the localization annotations of 264 proteins involved in functional modules and found 20 proteins that have not been annotated their localization in GO database. Then, we chose five proteins that have available antibodies, which were significantly changed under ρ^0^ mitochondria. Specifically, ZCD1 in mitochondrial ribosome function, GPT2 and PYCR2 in amino acid metabolism, CTSD in proteolysis, and HSPBP1 in protein folding on *mt* surface modules were tested. We confirmed that all five proteins localized in mitochondria and merged perfectly with the red fluorescence of the proteins with *mt* signal sequence (Figure 6).

## Discussion

Mitochondrial dysfunction caused by *mt*DNA damage is involved in many diseases and is likely to be a driving force behind aging and apoptosis [34]. Here, we investigated the systemic alterations of *mt* protein expressions by using a data-integrative network analysis. Respiratory-deficient ρ^0^ cells have been studied to characterize retrograde signaling, which is a controlling mechanism of information flow from the mitochondria to the nucleus and cytoplasm [35], but the systems property of dysfunctional *mt* state has not been studied extensively. Through network clustering analysis, we discovered that cells respond to pathological conditions by modulating the coordinated expression/transport of *mt* proteins.

Using a network analysis of proteomics data, we were able to find modules reflecting differentially regulated functions between normal and dysfunctional *mt* states. We found that up-regulated and down-regulated proteins of dysfunctional *mt* states were organized into two predominant subnetworks that exhibited distinct biological processes. It has been suggested that proteins cooperate with other proteins as a part of functional module which tend to physically associated or share similar expressions to deliver a certain biological function [36], [37]. Network analyses of molecular pathways or complexes elucidated the collective behavior of differentially expressed proteins and provided complementary information to conventional single gene-based analysis which routinely performed in proteomic analyses [38], [39]. We discovered that not only the relevant proteins changed their expression under dysfunctional *mt* state, but also the subnetworks composed of multiple functional protein groups changed their expression cooperatively to regulate biological processes. These network-module alterations are particular importance for relating an altered phenotype with dysfunctional *mt* state at molecular level because phenotypic alterations are more closely related with pathway remodeling than individual gene expression changes [38].

We found that functional modules controlling *mt* protein translation, folding, proteolysis, and *mt*DNA repair were up-regulated in the dysfunctional ρ^0^ state (Figure 3). To properly respond to changes in cellular states, these processes may require retrograde regulation [35]. Retrograde signaling regulates many cellular activities under pathophysiological conditions by changing the protein inventories of subcellular organelles. It may be possible that up-regulated *mt* proteins shuttle between the mitochondria and other organelles for intracellular communication [40]. Indeed, we found that the proteins of up-regulated functional modules tended to have multiple annotations of subcellular localization and to be involved in processes occurring inside and outside of mitochondria (Table S1). For example, AP-endonuclease 1 (APEX1) of the DNA repair module is known to shuttle from mitochondria to the nucleus in response to oxidative stress [41]. Heterogeneous nuclear ribonucleoprotein K (HNRPK), a member of the ribonucleoprotein complex associated with *mt*DNA transcription, has been detected in the nucleus, cytoplasm, and mitochondria [42]. These observations suggest that up-regulated functional modules act as cross-talk components connecting mitochondria with other organelles to sense and propagate the retrograde signal upon *mt*DNA depletion stress to facilitate mitochondrial survival.

We observed that up-regulated and down-regulated functional modules contain a small fraction of proteins with opposite protein expression patterns (Figure 3). On average, levels of 5.5% of the proteins in down-regulated functional modules were increased, while levels of 4.7% of the proteins in up-regulated functional modules were decreased under the dysfunctional ρ^0^ state. Proteins with opposite expression patterns in the functional module could act as negative regulators of the module [43]. For example, in the TCA cycle module, acetolactate synthase (AHAS) was up-regulated, while other proteins were down-regulated. It has been reported that upregulation of AHAS decreases the activity of the TCA cycle by reducing pyruvate flow into the cycle [44]. In addition, ATP6V1B2 and ATP6V1G1 in the ATP synthesis module were up-regulated, while other proteins were down-regulated. Upregulation of these proteins is known to participate in ATP hydrolysis, leading to the downregulation of ATP synthesis [45]. Moreover, two heat shock proteins, HSP90AA1 and HSP90AB1, involved in protein folding on the *mt* surface were down-regulated, while other proteins were up-regulated. The heat shock proteins regulate chaperone activity in response to ATP concentrations in the cell [46]. Under the dysfunctional ρ^0^ state, ATP reduction could induce the disruption of HSP90 chaperone activity. Thus, we suspect that oppositely expressed proteins may compensate for the function of other proteins in the same modules.

The expression of up- and down-regulated proteins was controlled in several different levels. From the comparisons between protein and mRNA abundance under the dysfunctional ρ^0^ state, we found that most up- and down-regulated *mt* proteins had increased mRNA expression levels (Figure 4). This indicated that up-regulated proteins were successfully recruited into ρ^0^ mitochondria. It has been proposed that there is a compensatory *mt* protein import pathway independent from the dissipated membrane potential [47]. This pathway is known to facilitate the translocations of proteins involved in *mt* protein folding and *mt*DNA repair to the mitochondria [48]. It implies that “emergency” proteins are increasingly imported to dysfunctional ρ^0^ mitochondria to repair pathophysiological *mt* conditions. The cross-talk properties of up-regulated functional modules support this idea.

One might expect that protein abundances in cytoplasm and mitochondria to be correlated with mRNA expression levels. However, we found that the functional modules of down-regulated proteins have up-regulated mRNA levels, suggesting that down-regulated proteins might experience difficulties in protein import into ρ^0^ mitochondria. To understand the differences of mRNA and protein expression of those proteins, we examined the levels of protein expression in ρ^0^ cytoplasm and mitochondria by using western blot analyses. The tested proteins, GLUD1, GTPBP3, ATP5A1, TFAM, and NDUFS6, were selected from each down-regulated functional module. We found that the cytoplasmic protein levels of GLUD1 (amino acid metabolism), GTPBP3 (tRNA metabolism), and ATP5A1 (ATP synthesis) were increased, whereas the mitochondrial levels of those proteins were found to be decreased (Figure S3), suggesting that those proteins remained in the cytoplasm and did not properly transport into mitochondria. Meanwhile, both cytoplasmic and mitochondrial protein levels of TFAM (*mt*DNA replication) and NDUFS6 (electron transport) were decreased (Figure S3). It might be possible that these proteins degraded more rapidly than synthesized in ρ^0^ cell, consequently showed down-regulated protein expression levels.

Mitochondrial protein compositions change to remodel the organization of *mt* protein functional networks in response to changes in cellular states. Analysis of *mt* protein functional networks elucidated the biological implications of *mt* regulatory mechanisms under dysfunctional *mt* states. Our efforts of systematic data-integrative analysis to evaluate the reliability of proteomics data and to identify important functional modules of mitochondrial proteins can be valuable to computational biology community working on gene expression and proteomics analysis. First, we applied a data-integrative approach to select protein list by using various databases, proteomics datasets, and protein functional network. Mitochondrial proteins were organized into functional modules to identify significantly altered biological processes under different cellular states. The framework of our systematic data-integrative analysis may be useful to reliable proteome analyses for other cellular systems. Second, organizing the thousand mitochondrial proteins into groups of up- or down-regulated ones and identifying functional modules are necessary steps in getting an in-depth understanding of the complex molecular mechanism of mitochondria. Third, mapping both mitochondrial proteins and mRNA expression information together is critical to understand the cooperative expression regulation of mitochondrial functional modules. Such multi-dimensional data analysis can be a valuable asset to develop novel system level models and methods. Also, experimental biologists can utilize our dataset as a resource for target selection to elucidate regulatory mechanisms of *mt* proteins under dysfunctional *mt* states.

## Materials and Methods

### Cell culture and mitochondria isolation

Human *mt*DNA-depleted (ρ^0^) 143B TK^−^ osteosarcoma cells and parental normal ρ^+^ cells (provided from Dr. Wei YH, National Yang-Ming Univ., Taipei, Taiwan) were cultured in high glucose (4.5 g/L) Dulbecco's Modified Eagle's Medium (DMEM) supplemented with 10% fetal bovine serum (FBS), antibiotics (100 µg/ml penicillin/streptomycin mix), and uridine (50 µg/ml) in a humidified atmosphere at 37°C with 5% CO_2_ as described [49]. The absence of *mt*DNA in ρ^0^ cells was verified by PCR and PicoGreen staining (Figure S4). PicoGreen is a sensitive staining dye to visualize *mt*DNA [50]. The PCR primers were 5′-TTC CAC ACA GAC ATC ATA AC-3′ and 5′-CCT ATT TGT TTA TGG GGT GA-3′ for *mt*DNA (410 bp at 55°C for 21 cycles) and 5′-TTC TAC AAT GAG CTG CGT GTG GCT-3′ and 5′-GCT TCT CCT TAA TGT CAC GCA CGA-3′ for β-actin (378 bp, 65°C for 27 cycles). Meanwhile, cells were treated with PicoGreen (3 µl/ml) for 1 h, washed with DPBS, and stained with 100 nM Mitotracker-Orange (Mito-T, Molecular Probes) for 10 min. Then, cells were fixed with 4% paraformaldehyde for 20 min and observed via confocal microscopy (Leica TCS-SP2).

Pure mitochondria from ρ^0^ and ρ^+^ cells (2×10^8^) were prepared by ultracentrifugation by using 30–50% (1.1 and 1.6 g/ml) Optiprep™ density gradient media (Sigma-Aldrich) as described previously [51]. The purity of mitochondria was confirmed by western blot analysis. *Mt* proteins, COX I, COX IV, and HSPD1 (HSP60), exhibited significant reductions in levels in ρ^0^ mitochondria compared to their levels in ρ^+^ mitochondria, supporting complete mtDNA depletion and good mt preparation. Meanwhile, nuclear fraction marker (HDAC1), cytoplasmic fraction marker (NF-κB and SOD1), ER fraction marker (GRP78), and lysosomal marker (LAMP1) were not detected in both ρ^0^ and ρ^+^ mitochondria (Figure S5). β-actin was used for equal loading verification. Then, we applied quantitative cICAT analysis to compare protein abundances in ρ^0^ and ρ^+^ mitochondria.

### Analysis of human *mt* proteomics data

To validate whether the proteins identified from cICAT were associated with mitochondria, we examined the GO cellular component annotation of the proteins identified from proteomics. Then, we performed a systematic data-integrative analysis to examine whether proteins identified from proteomics were matched with 13 reference *mt* protein datasets representing complementary independent studies of *mt* proteome, which include seven *mt* protein databases (HMPDb (http://bioinfo.nist.gov/hmpd/index.html), Maestro [52], MitoProteome [53], MitoRes [54], Locate [55], MitoP2, and SVM-trained MitoP2 [56]) and six *mt* proteomics datasets (two MitoCarta datasets [57], and four *mt*DNA depleted mitochondria proteomics datasets [33], [58], [59], [60]). Some of the proteins from the database might contain mitochondrial proteins from other species, thus we only selected human *mt* proteins from these datasets to construct a reliable *mt* protein list. We validated 569 human proteins annotated as mitochondrial proteins and observed from at least one reference mitochondrial protein dataset.

Next, the Human protein reference database (HPRD) [61] and Search Tool for the Retrieval of INteracting Genes/proteins (STRING) database [62] were utilized for detecting physical and functional associations of the resulting proteins. STRING provides a large set of known and predicted protein-protein associations by compiling experimental repositories, curated pathway database, literature-mining resources, and computational predictions. Two proteins were connected in the STRING network if they shared a substrate in a metabolic pathway, co-expressed, co-regulated, connected by protein-protein interactions, or involved in the same protein complex. We additionally identified 201 proteins, of which 82 proteins were physically associated and 119 proteins were functionally associated with the first 569 *mt* proteins. A total of 770 proteins were defined as reliable *mt* proteins.

### Construction of human *mt* protein functional network and identification of functional modules

We constructed the human *mt* protein functional network using physical and functional link information extracted from the STRING database (ver. 8.0) with the 770 reliable *mt* proteins. The resulting *mt* protein functional network contained 726 proteins with 13,618 links (Figure 2A), which included 4,854 intraregulatory links and 859 interregulatory links (Figure 2B). Intraregulatory links were defined as the interactions among up-regulated proteins or among down-regulated proteins, whereas interregulatory links were the interactions among up-regulated proteins and down-regulated proteins.

We separated the mitochondrial protein functional network into functional modules (Figure 1A). A functional module was determined by examining whether a group of proteins was physically connected or functionally linked. To identify modules, we used hierarchical average-linkage clustering by using the OC software (http://www.compbio.dundee.ac.uk/Software/OC/oc.html) with the STRING confidence scores as a similarity measure. STRING confidence score represented the probability of finding the proteins which were related by one or more cellular and genetic interactions. Then, we assigned biological function to the module by using Ontologizer 2.0, which collects GO representative functional annotations from proteins in each module [63], and selected functional modules if the significance (*p*-value) of GO enrichment was less than 0.01. The enrichment of up- or down-regulated proteins in a given functional module was used to check the consistency of protein expression in the module (hypergeometric tests, *p*-value <0.01). Finally, we identified 13 functional modules consisting of five up-regulated and eight down-regulated modules.

### Microarray analysis

Total RNA from ρ^0^ or ρ^+^ cells was isolated using TRIzol (Invitrogen) and quantified with a NanoDrop spectrophotometer (NanoDrop Technologies, Inc.) (n = 3). Microarray analysis was performed in triplicate by using the Illumina Sentrix HumanRef-8 Expression BeadChip according to the Illumina Bead Array Technical Manual. Briefly, total RNA (500 ng) was used for cDNA synthesis, followed by an amplification/labeling step (in vitro transcription) to synthesize biotin-labeled cRNA by using the Illumina® TotalPrep RNA amplification kit (Ambion, Inc.). The cRNA sample (750 ng) was hybridized to the BeadChip and stained with streptavidin-Cy3. The chips were dried and scanned by the BeadArray reader. The raw scan data were subjected to logarithmic transformation (log_2_ ratios of fluorescence intensities) and quantile normalization by using the Avadis 4.3 software (Strand Genomics). Statistical significances were adjusted by the Benjamini-Hochberg FDR multiple-testing correction. Genes were filtered out by using the detection *p*-value threshold (*p*>0.05).

All microarray data reported in this study are described in accordance with MIAME guidelines and have been deposited in the National Center for Biotechnology Information Gene Expression Omnibus (GEO, http://www.ncbi.nlm.nih.gov/geo/) public repository, and they are accessible through GEO accession (GSE22970).

### Protein identification and quantification

For cICAT labeling, protein extracts from the pure ρ^0^ or ρ^+^ mitochondria were prepared with lysis buffer (10 mM HEPES, pH 7.9, 10 mM KCl, 2 mM MgCl_2_, 0.5 mM dithiothreitol, 1 mM phenyl-methylsulfonyl fluoride [PMSF]). Proteins were concentrated, and other non-protein materials such as salts were removed by the acetone precipitation method. Precipitated proteins were denatured in labeling buffer (6 M urea, 0.05% SDS, 5 mM EDTA, 50 mM Tris-Cl, pH 8.3) for 30 min and reduced with 5.3 mM Tris (2-carboxyethyl) phosphine hydrochloride (TCEP) for 30 min at 37°C. After readjustment to pH 8.3 with 1 M Tris-Cl buffer (pH 8.3), the ρ^+^ or ρ^0^ protein samples were labeled with a 15-fold molar excess of cleavable cICAT light (^12^C) or heavy (^13^C) reagents (Applied Biosystems, Foster City, CA) relative to proteins for 2 h at 37°C. Each 110-µg aliquot of separately labeled samples was equally combined and digested by trypsin (Promega, Madison, WI). The cICAT-labeled peptides were selectively isolated by strong cation exchange (SCX) and avidin affinity chromatography on a manually programmed AKTA Explorer 100 system (Amersharm Pharmacia, Sweden). Biotin moieties from cICAT-labeled peptides were cleaved by incubation for 1.5 h with 95% trifluoroacetic acid at 37°C. Samples were then dried in a speed-vacuum dryer and dissolved with 0.4% acetic acid for LC-MS/MS analysis. A schematic summary of the cICAT analysis workflow is presented in Figure S1.

The cICAT-labeled peptides were loaded on a nanospray tip coupled to a capillary reverse-phase column (14 cm×75 µm, Magic C18aq; Michrom BioResources, Auburn, CA) packed in-house by using a helium pressure cell. Peptides were eluted with a linear gradient of 5–35% buffer B (running buffer A: 0.1% formic acid in H_2_O; elution buffer B: 0.1% formic acid in 100% acetonitrile) over 90 min at 200 nl/min using an Agilent 1100 capillary pump system. Eluting peptides from the column were analyzed using LTQ linear ion trap mass spectrometers (Thermo Finnigan, San Jose, CA). A MS survey scan from 300–2000 m/z was acquired with three μscans followed by three data-dependent MS/MS scans (isolation width, 1.5 m/z; normalized collision energy, 28%; dynamic exclusion lists, 100; dynamic exclusion duration, 3 min). Data from RAW MS/MS files (minimum ion counts, 15; minimum peak intensity threshold, 1; mass range, 600–4300 Da) were generated, and peptide sequences from the data were assigned against the International Protein Index (IPI) human database (Version 3.13; 57,034 proteins) including known contaminants (180 entries) by using a 4-node SEQUEST (version 27, revision 9) cluster with the following search parameters: specificity (no enzyme), number of missed cleavage (max three sites), cysteine mass (fixed, +227.13 for light cICAT), cysteine mass (variable, +9 for heavy cICAT), methionine mass (variable, +16 for oxidation), mass tolerance of precursor ion (3.0) and fragment ion (0.5), mass type of precursor and fragment ion (average mass), and subsequence (cysteine residue). To discriminate true proteins from false positives and to quantify the abundance of proteins, we used Trans-Proteomic Pipeline of Institute for Systems Biology (TPP; Version 1.7.2; INTERACT, PeptideProphet™, ProteinProphet™, XPRESS programs). The peptides with P≥0.50 by PeptideProphet™ were applied to ProteinProphet™. The proteins with P≥0.95 by ProteinProphet™ were considered to have the correct identification. Single- and double-hit proteins of the correct identifications were further validated through manual inspections of MS/MS spectra (false positive rate, below 0.4%). Quantification of peptides and proteins was performed with XPRESS software, and the peptides with bad quality (e.g., below S/N≤2) were not considered by quantification. Finally, we identified nuclear-encoded 1,121 *mt* proteins that included 313 down-regulated proteins (ρ^0^/ρ^+^≤0.67), 201 up-regulated proteins (ρ^0^/ρ^+^≥1.5), and 607 not significantly changed (0.67<ρ^0^/ρ^+^<1.5) proteins. The thresholds of 0.67 represents the 1.5-fold decrease (ρ^0^/ρ^+^≤1/1.5), whereas the threshold of 1.5 represents 1.5-fold increase (ρ^0^/ρ^+^≥1.5). Protein abundance ratio smaller than 0.67 or larger than 1.5 were routinely-applied thresholds indicating significant changes in proteomic analyses [64], [65], [66], [67]. See Table S1 for further details on the list of identified *mt* proteins.

### Western blot analysis

Cells were lysed with lysis buffer (50 mM Tris HCl, pH 7.5, 0.1 M NaCl, 1 mM EDTA, 1% Triton X-100, 10 µg/ml each of aprotinin and leupeptin, and 1 mM PMSF). In some cases, mitochondrial fractions were lysed with the lysis buffer. A portion of cells (20–30 µg) or mitochondrial lysates (10 µg) were separated on 12% SDS-PAGE, transferred onto nitrocellulose membranes (Schleicher and Schuell), and subjected to western blot analysis by using designated primary antibodies. Horseradish peroxidase-conjugated secondary antibodies (Cell Signaling Technology, Beverly, MA) followed by ECL (Amersham Biosciences Inc., Piscataway, NJ) were used for detection.

Antibodies against APEX1, STUB1, and ATP5A1 were purchased from Santa Cruz Biotechnology (Santa Cruz, CA). GTPBP3 and HDAC1 antibodies were purchased from Abcam (Cambridge, UK), and anti-eIF4A1 was obtained from Cell Signaling Technology (Danvers, MA). Antibodies against ALDH2, ALDH6A1, MTRF1, NDUFS6, HNRPM, GLUD1, and PLG were purchased from Abnova (Taipei, Taiwan). Antibodies against COXI and COXIV were purchased from Invitrogen (Karlsruhe, Germany), and TFAM were prepared in our laboratory [51]. PARP1, HSPD1 (HSP60), NF-κB, SOD1, XRCC5 (Ku80), XRCC6 (Ku70), and β-actin were purchased from Santa Cruz Biotechnology.

### Localizations of GFP fusion proteins

SK-Hep1 cells were stably transfected with pDsRed2-mito vectors (Clontech) containing the *mt* signal sequence of COXVIII in front of red fluorescent protein (Red2). The cDNAs of several candidate proteins were synthesized from the total RNA of ρ^+^ cells by RT-PCR, sub-cloned into the T-easy vector (Promega), and then cloned into the N-terminus of the pEGFP-N3 vector (BD Bioscience). The resulting GFP-hybrid plasmids of pZCD1-EGFP, pGPT2-EGFP, pPYCR2-EGFP, pCTSD-EGFP, and pHSPBP1-EGFP were transfected using Superfect (QIAGEN, Valencia, CA) into DsRed2-mito-SK-Hep1 cells. At 48 h post-transfection, the transfected cells were fixed with 4% paraformaldehyde, mounted, and imaged using a confocal microscope (Carl Zeiss LSM 5). The *mt* localizations of candidate proteins were determined by the overlap of EGFP and DsRed signals.

## Supporting Information

Figure S1 **Workflow of comparative cICAT analysis of mitochondria proteomes.** See Materials and Methods for details.
(TIF)

Figure S2 **The reproducibility of the changes in protein abundances detected by cICAT quantification.** Thirty-three *mt* proteins were observed from both cICAT and 2DE proteome datasets. Similar expression patterns between our *mt* proteomics data (black) and 2DE analysis (light gray) are shown.
(TIF)

Figure S3 **The level of protein expression in cytoplasm and mitochondria.** Western blot analysis of five proteins in cytosolic and mitochondrial fractions isolated from ρ^+^ and ρ^0^ cells. β-actin was used as a loading control.
(TIF)

Figure S4 **Confirmations of** ***mt*** **DNA depletion in ρ^0^ cells.** (A) PCR amplification of *mt*DNA. Genomic DNAs isolated from ρ^+^ or ρ^0^ cells were utilized as templates to amplify *mt*DNA and nuclear DNA-encoded β-actin control. (B) Cellular nucleotide staining. Cells were treated with PicoGreen for 1 h, washed with DPBS, and then stained with Mitotracker orange (Mito-T, 100 nM) for 10 min. The cells were fixed with paraformaldehyde for 20 min and visualized by conformal microscopy (×1000). *mt*DNA was observed only in ρ^+^ cells.
(TIF)

Figure S5 **Identification of purified mitochondria.** Mitochondria were isolated using gradient-based ultracentrifugation as described. Proteins from a total lysate (30 µg), and mitochondria (10 µg) were resolved using 12% SDS-PAGE and analyzed by western blot. Antibodies against the following marker proteins were used: COXI, COXIV, and HSPD1 (HSP60) for mitochondria, HDAC1 for nucleus, NF-κB and SOD1 for cytoplasm, GRP78 for ER, and LAMP1 for lysosome. β-actin served as a loading control.
(TIF)

Table S1 List of the 1,121 proteins identified by cICAT.
(XLS)

Table S2 List of enriched functions in up-regulated *mt* proteins or down-regulated *mt* proteins.
(XLS)

Table S3 List of up-regulated functional modules and down-regulated functional modules.
(XLS)

Table S4 Comparisons between protein and mRNA expression.
(XLS)
