# Supplementary figures and images for: Network Clustering Revealed the Systemic Alterations of Mitochondrial Protein Expression
Source: PLoS Comput Biol. 2011 Jun 30;7(6):e1002093. doi: 10.1371/journal.pcbi.1002093 (PMC3127811; doi:10.1371/journal.pcbi.1002093)

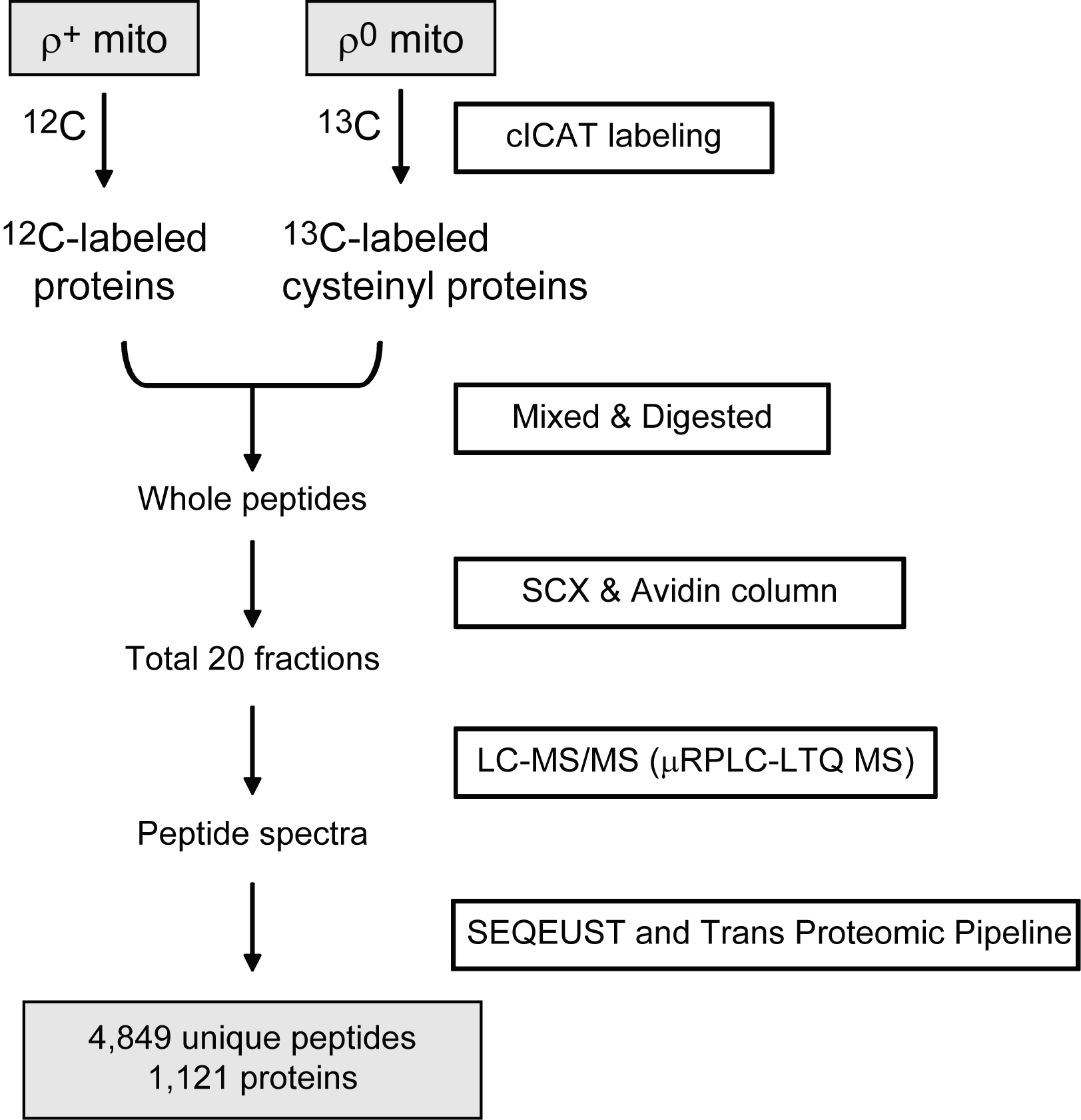

Supplement: Figure S1 — Workflow of comparative cICAT analysis of mitochondria proteomes. See Materials and Methods for details. (TIF) [file pcbi.1002093.s001.tif]

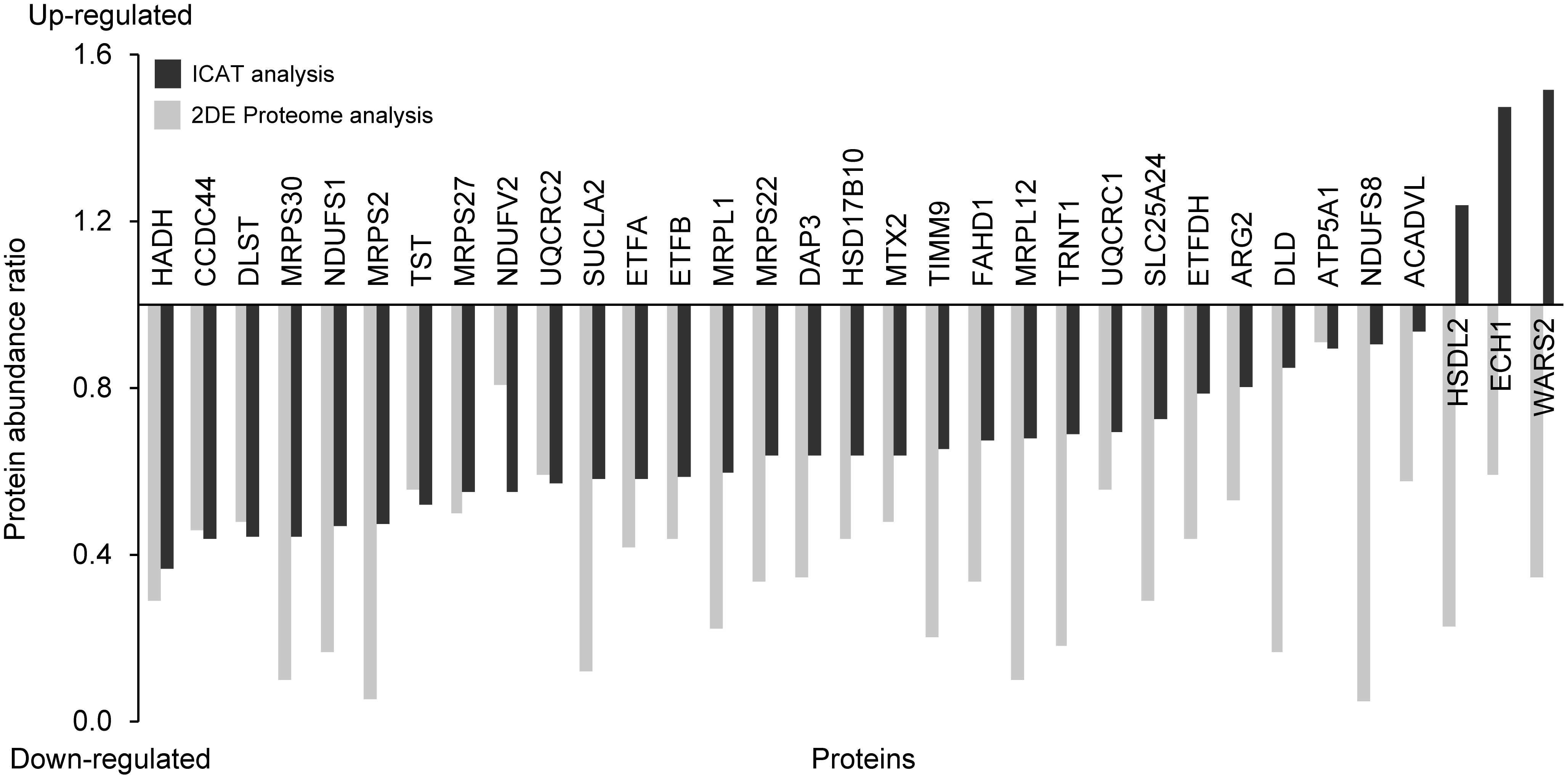

Supplement: Figure S2 — The reproducibility of the changes in protein abundances detected by cICAT quantification. Thirty-three mt proteins were observed from both cICAT and 2DE proteome datasets. Similar expression patterns between our mt proteomics data (black) and 2DE analysis (light gray) are shown. (TIF) [file pcbi.1002093.s002.tif]

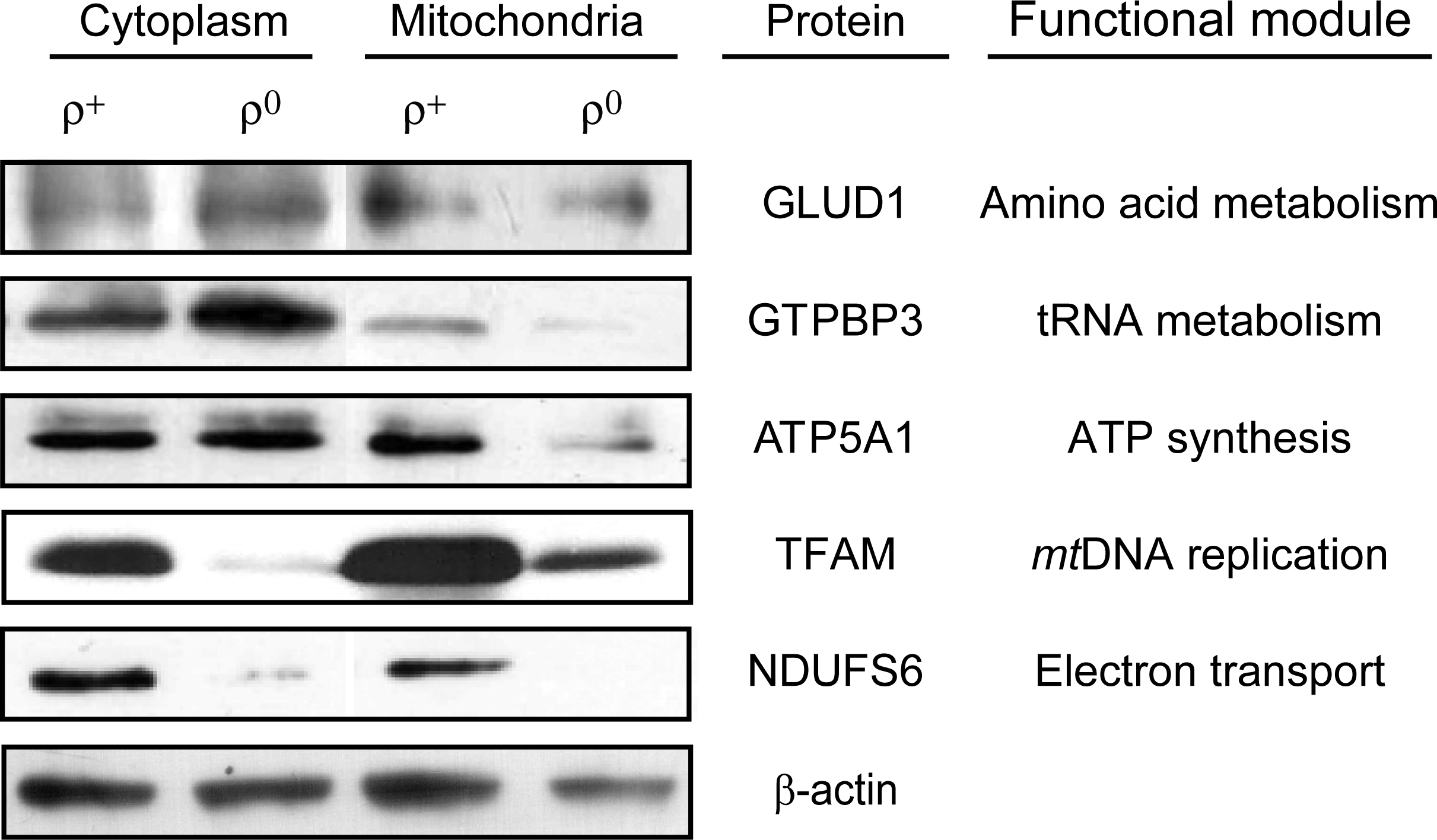

Supplement: Figure S3 — The level of protein expression in cytoplasm and mitochondria. Western blot analysis of five proteins in cytosolic and mitochondrial fractions isolated from ρ+ and ρ0 cells. β-actin was used as a loading control. (TIF) [file pcbi.1002093.s003.tif]

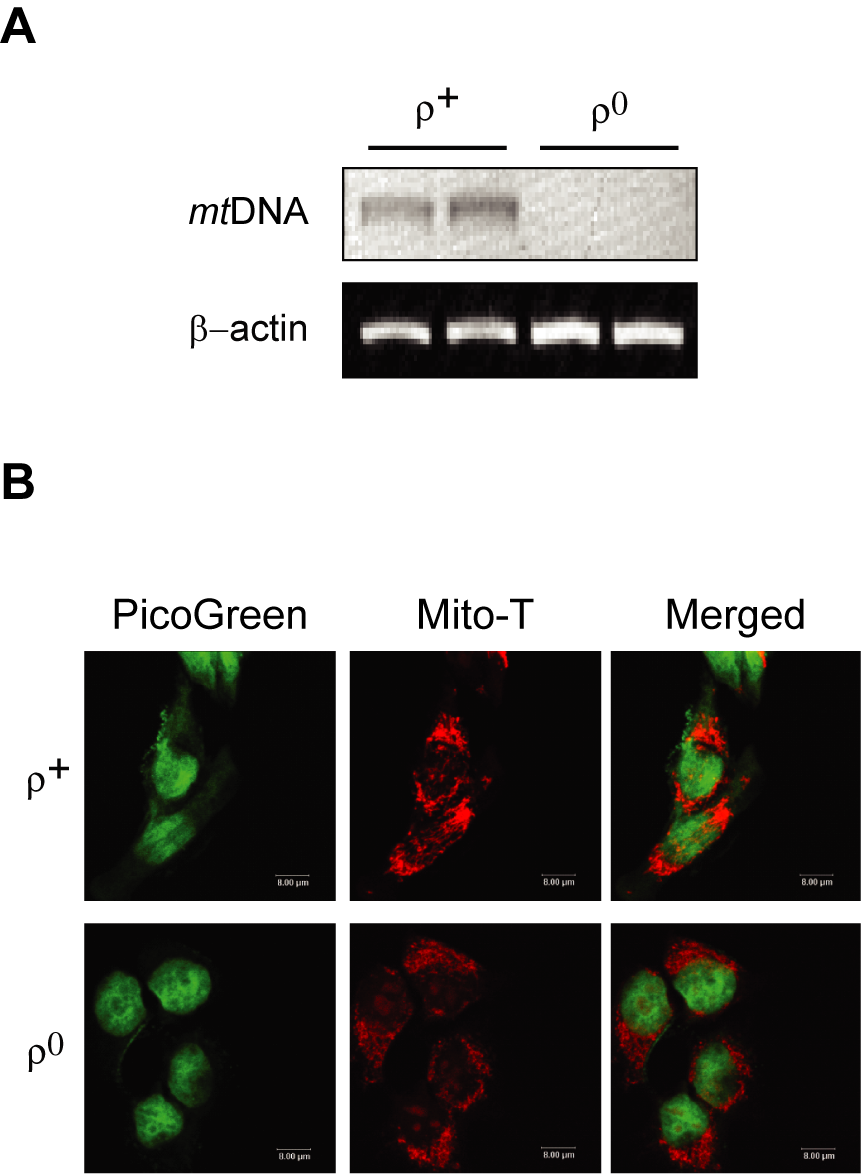

Supplement: Figure S4 — Confirmations of mt DNA depletion in ρ0 cells. (A) PCR amplification of mtDNA. Genomic DNAs isolated from ρ+ or ρ0 cells were utilized as templates to amplify mtDNA and nuclear DNA-encoded β-actin control. (B) Cellular nucleotide staining. Cells were treated with PicoGreen for 1 h, washed with DPBS, and then stained with Mitotracker orange (Mito-T, 100 nM) for 10 min. The cells were fixed with paraformaldehyde for 20 min and visualized by conformal microscopy (×1000). mtDNA was observed only in ρ+ cells. (TIF) [file pcbi.1002093.s004.tif]

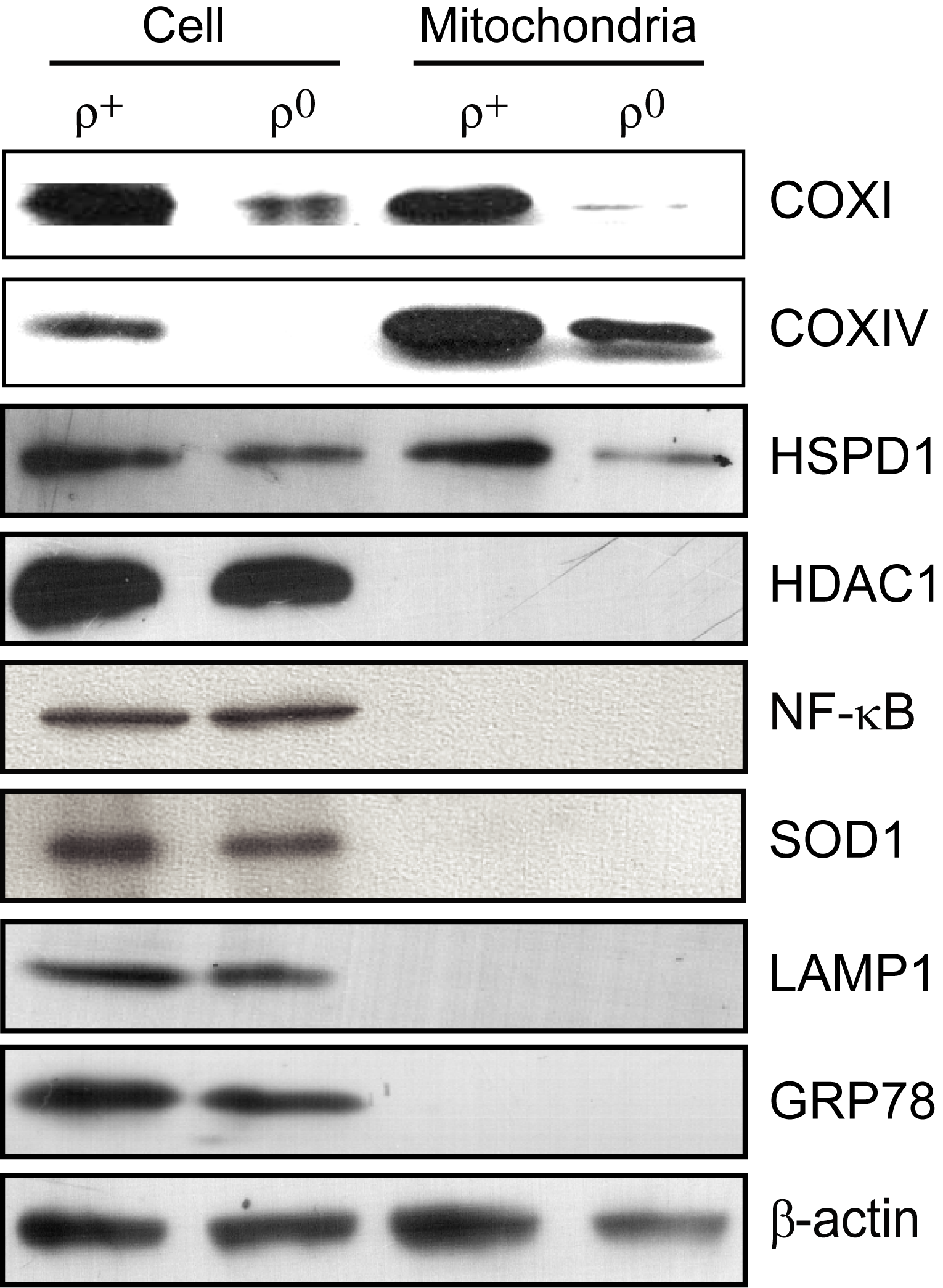

Supplement: Figure S5 — Identification of purified mitochondria. Mitochondria were isolated using gradient-based ultracentrifugation as described. Proteins from a total lysate (30 µg), and mitochondria (10 µg) were resolved using 12% SDS-PAGE and analyzed by western blot. Antibodies against the following marker proteins were used: COXI, COXIV, and HSPD1 (HSP60) for mitochondria, HDAC1 for nucleus, NF-κB and SOD1 for cytoplasm, GRP78 for ER, and LAMP1 for lysosome. β-actin served as a loading control. (TIF) [file pcbi.1002093.s005.tif]
